# From Stock Bottle to Vaccine: Elucidating the Particle Size Distributions of Aluminum Adjuvants Using Dynamic Light Scattering

**DOI:** 10.3389/fchem.2016.00048

**Published:** 2017-01-09

**Authors:** Emma Shardlow, Matthew Mold, Christopher Exley

**Affiliations:** Lennard-Jones Laboratories, The Birchall Centre, Keele UniversityStaffordshire, UK

**Keywords:** aluminum adjuvants, vaccine characterization, particle size, zeta potential, antigen-adjuvant complex

## Abstract

The physicochemical properties of aluminum salts are key determinants of their resultant adjuvanticity *in vivo* when administered as part of a vaccine. While there are links between particle size and the efficacy of the immune response, the limited literature directly characterizing the PSD of aluminum adjuvants has stymied the elucidation of such a relationship for these materials. Hence, this comparative study was undertaken to monitor the PSD of aluminum adjuvants throughout the process of vaccine formulation using DLS. A significant proportion of the stock suspensions was highly agglomerated (>9 μm) and Alhydrogel® exhibited the smallest median size (2677 ± 120 nm) in comparison to Adju-Phos® or Imject alum® (7152 ± 308 and 7294 ± 146 nm respectively) despite its large polydispersity index (PDI). Dilution of these materials induced some degree of disaggregation within all samples with Adju-Phos® being the most significantly affected. The presence of BSA caused the median size of Alhydrogel® to increase but these trends were not evident when model vaccines were formulated with either Adju-Phos® or Imject alum®. Nevertheless, Alhydrogel® and Adju-Phos® exhibited comparable median sizes in the presence of this protein (4194 ± 466 and 4850 ± 501 nm respectively) with Imject alum® being considerably smaller (2155 ± 485 nm). These results suggest that the PSD of aluminum adjuvants is greatly influenced by dilution and the degree of protein adsorption experienced within the vaccine itself. The size of the resultant antigen-adjuvant complex may be important for its immunological recognition and subsequent clearance from the injection site.

## Introduction

Where vaccines contain recombinant antigens, which are typically weakly immunogenic in their own right, it is often necessary to also include an adjuvant within the formulation itself. Adjuvant compounds increase the efficacy and longevity of the immunological response through potentiation and polarization, while having the advantage that they themselves are not expected to be individually antigenic (Cox and Coulter, 1997; Gupta, 1998). Despite the recent advances in vaccinology only a limited number of materials are currently used within clinical immunizations and aluminum salts still remain the most popular (Reed et al., 2013), perhaps owing to their perceived superior safety record and low production cost (Goldenthal et al., 1993; Lindblad, 2004). The lack of cell-mediated immunopotentiation and associated weak adjuvanticity of these materials (Grun and Maurer, 1989; Brewer et al., 1996; Jordan et al., 2004), however, is increasingly problematic for vaccinologists as are the numerous reports of adverse reactions observed in individuals inoculated with aluminum-containing vaccines (Gherardi et al., 2001; Shaw and Petrick, 2009; Shoenfeld and Agmon-Levin, 2011). Nevertheless, aluminum salts continue to provide an archetypal model for the study of both immunological and mechanistic events following immunization (Exley et al., 2010).

The degree of antigenic adsorption at the adjuvant colloidal interface is generally regarded as a major contributor to their adjuvanticity in biological systems and is thus an important consideration in vaccine design (Gupta, 1998). Where strong adsorption coefficients are observed either through ligand exchange or favorable electrostatic interactions, enhanced antigenic retention at the site of inoculation appears to be the most probable outcome (Iyer et al., 2003, 2004; Noe et al., 2010). This is particularly beneficial with regards to the subsequent recognition and uptake of material by infiltrating phagocytes (Mannhalter et al., 1985; Morefield et al., 2005), which in the case of aluminum is required for the inflammasome-mediated release of pro-inflammatory cytokines (Eisenbarth et al., 2008). Adsorptive parameters, however, require careful optimization and several studies have shown that the strength of antigenic adsorption is in fact inversely proportional to the antibody response mounted *in vivo* (Iyer et al., 2003; Hansen et al., 2007, 2009; Noe et al., 2010).

Of the many factors that are known to influence both protein adsorption and the immunogenicity of vaccines, particle size is the parameter that has received the least attention. There is some evidence to suggest that the size of aggregates within simulant vaccines is influenced by the extent of proteinaceous adsorption experienced at the surface of the adjuvant material (Morefield et al., 2005). Indeed, the authors revealed that formulations where strong attractive associations were present between vaccine components contained larger entities than those lacking these interactions when administered into biological fluid. Recent studies have also revealed that a small elevation in the particle size of the native adjuvant significantly impedes protein adsorption through a reduction in the adsorptive capacity of the material, although this has only been demonstrated using a single commercial preparation (Huang and Wang, 2014). A limitation of these studies, however, is the lack of data comparing the size of the antigen-complex with that of the adjuvant itself. Furthermore, only a limited number of communications refer directly to the size of native aluminum salts and the majority of these report average core sizes obtained under the anhydrous conditions of TEM (Burrell et al., 2000a; Harris et al., 2012; Shah et al., 2014).

This study was therefore undertaken as a cross-comparative insight into the hydrodynamic particle size distribution (PSD) of aluminum adjuvants and how this parameter was influenced upon exposure to the myriad environments associated with the process of vaccine formulation.

## Experimental

### Materials

Stock solutions of the commercial adjuvants Alhydrogel® (aluminum oxyhydroxide) and Adju-Phos® (aluminum hydroxyphosphate) were obtained from Brenntag Biosector, Denmark and contained 10.1 and 5 mg/mL Al respectively. A research preparation (Imject alum®) was procured from Pierce, UK and contained 40 mg/mL of aluminum and magnesium hydroxide (*ca* 13.3 mg/mL Al). Bovine serum albumin (BSA) was purchased from Sigma Aldrich, UK.

### Formulation of simulant vaccines

Vaccine simulants without antigen were prepared from stock adjuvant preparations which were diluted into physiological saline (9 g/L) to achieve a final aluminum concentration of *ca* 250 μg/mL. The final adjusted pH of these preparations was 7 ± 0.1.

Model vaccines containing BSA were prepared from a stock solution containing 1 mg/mL protein in physiological saline (pH 7 ± 0.1). Adjuvant stocks were added dropwise to saline solutions containing *ca* 50 μg/mL BSA to achieve a final aluminum concentration of *ca* 250 μg/mL and re-adjusted to pH 7 ± 0.1 using 0.1 M NaOH.

Both simulants and model vaccines were subject to gentle agitation using a magnetic stirrer at 25°C for a period of 1 h in order to allow the latter adequate time for protein adsorption to occur before analysis.

### Dynamic light scattering

Particle size characterization was performed via photon correlation spectroscopy using a Zetasizer Nano ZS (Malvern Instruments, UK) equipped with a 633 nm laser, which collected scattered light at an angle of 173°. Samples were introduced into pre-rinsed polystyrene cuvettes and size determinations were performed over a range of 0.6–10 μm at 25°C. All samples had an automatic attenuation setting of 5–7 and a count rate which greatly exceeded that determined through the analysis of the pre-filtered sodium chloride diluent (50 kcps).

A total of five measurements were made per sample replicate and only measurements that generated a multimodal fit error of <0.005 and a PDI < 0.7 were accepted to ensure the quality of the data collected (exclusive of data obtained for native adjuvant stocks). The average of these measurements was used to derive the size distribution values represented in the following experimental figures.

The hydrodynamic size of particles (Dh) was determined through the application of the Stokes-Einstein equation (Equation 1), where the translational diffusion coefficient (D) defines the velocity of random movement (Brownian motion) experienced via particulate components of the system.

(1) $$\begin{array}{l} \text{Dh }=\text{ kT}/3\pi \eta \text{D} \end{array}$$

The instrumentation generated two intensity based measures of size: One derived from a cumulant algorithm (*Z*-average) and another from a non-negative least squares fit of the correlation function (size distribution). Due to the multimodality of the size distributions obtained only the latter was used as part of the analysis. Despite the maximum limit of the instrumentation being specified as 10 μm, the distribution analysis is only capable of detecting particles up to *ca* 9 μm in size. As a consequence this value will be used as the upper maximum in the following results section.

### Zeta potential measurements

Zeta potential characterization was performed via electrophoretic light scattering using the same instrumentation as that used for the determination of particle size. Samples were introduced into polystyrene folded capillary cells containing gold plated beryllium/copper electrodes and five measurements were made per sample replicate.

### Transmission electron microscopy (TEM)

Vaccine preparations visualized by TEM were subject to the following protocol optimized by Mold et al. (2013). Samples were prepared upon pre-coated S162 200 mesh formvar/carbon coated copper grids (Agar Scientific, UK).

Grids were prepared through immersion in a 30 μL bead of freshly prepared sample for 2 mins, passed through ultrapure water, wicked and stained with 2% *w/v* uranyl acetate (in 70% *v/v* ethanol) for 30 s. Following staining, grids were re-wicked, passed through ultrapure water and placed into 30 μL 30% *v/v* ethanol for 30 s. Grids were finally re-wicked, covered and allowed to dry for up to 24 h, prior to analysis via TEM. Each grid was visualized using a JEOL1230 transmission electron microscope (operating voltage–100 kV) with a Megaview III digital camera attachment from Soft Imaging Systems (SIS). Captured images were analyzed using iTEM universal TEM imaging platform software.

### Statistical analysis

Statistical analysis of the data was performed using GraphPad Prism software (v.7). Multiple comparisons were performed using ANOVA followed by Tukey *post-hoc* tests where data satisfied tests for normality (Shapiro-Wilk where *p* ≥ 0.05). Datasets which violated these assumptions were analyzed using Kruskal-Wallis test followed by Dunn *post-hoc* tests. Pairwise comparisons were made using two-tailed unpaired *t*-tests or Mann–Whitney *U*-tests if normality assumptions were violated. A *p* value of ≤ 0.05 was accepted as statistically significant.

## Results

### The PSD and zeta potential of native aluminum adjuvants

**Figure 1:**
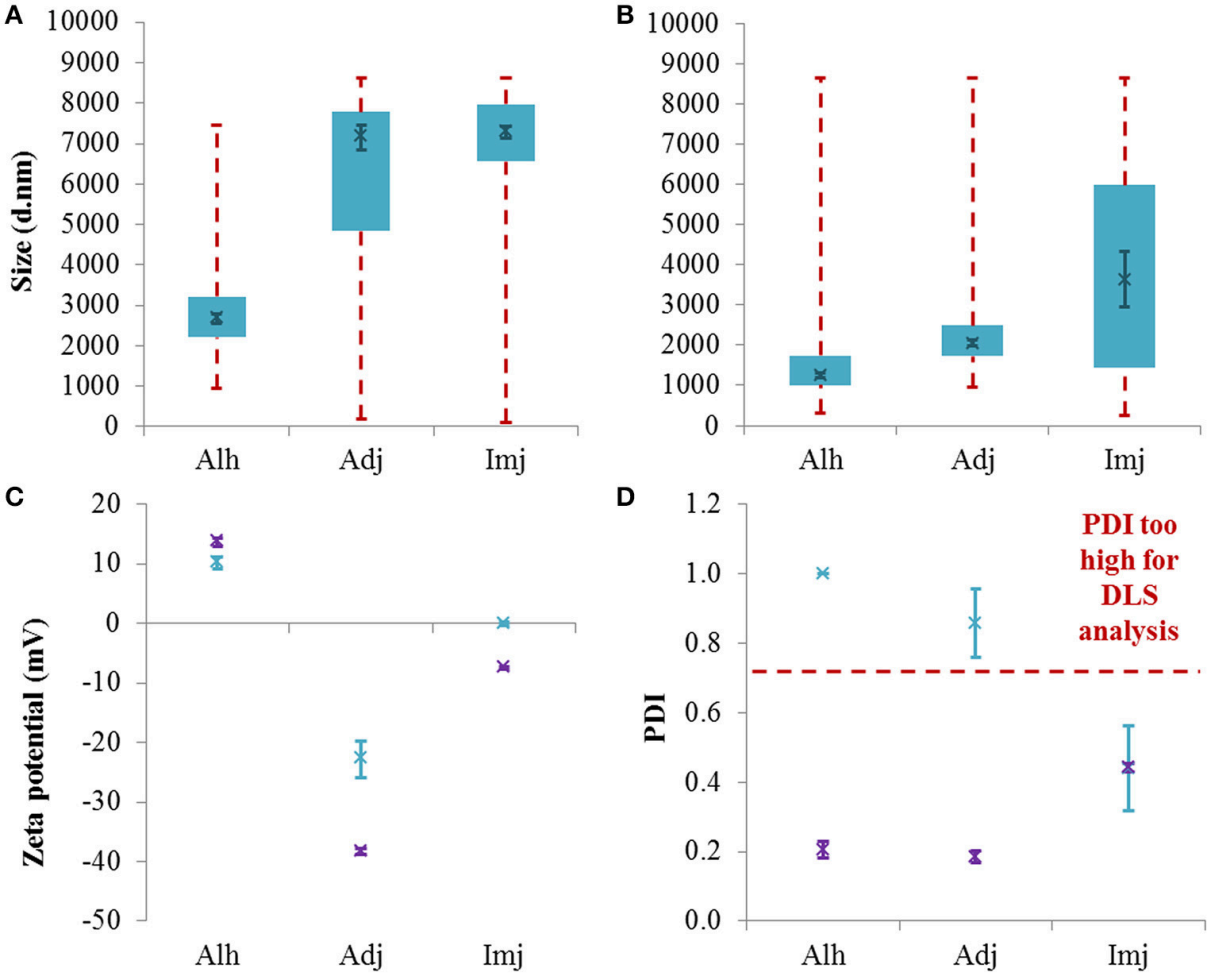
**Particle size distributions generated for native aluminum adjuvants (A)** and those diluted to 1 mg/mL Al in UPW **(B)**. Box plots are representative of the interquartile range of the data while red dashed lines indicate the maxima and minima. Solid green crosses represent the d50 distribution value (nm). Zeta potential **(C)** and PDI **(D)** data is also included where green and purple crosses represent native and diluted stocks respectively. Error bars represent the ±SEM of the measurement where *n* = 5.

In its native form, Alhydrogel® generated a broad monomodal distribution spanning *ca* 955–7456 nm with the majority of the scattering intensity being detected between *ca* 2.2–3.3 μm (Figure 1A). Meanwhile, the size distributions of Adju-Phos® & Imject alum® were characterized by several discrete populations between *ca* 100 and 8635 nm (Figure S1A) with those exhibiting the greatest scattering intensities located toward the maximum limit of the instrumentation. Relative comparisons inferred that the median size of Alhydrogel® particles was significantly smaller than either Adju-Phos® or Imject alum® (2677 ± 120, 7152 ± 308, and 7294 ± 146 nm respectively, *P* ≤ 0.0001); however, the PDI of the commercial adjuvants was consistently high and thus compromised the validity of this data (Figure 1D). Interrogation of the correlation coefficients obtained for each material also revealed a tendency toward sedimentation as demonstrated by the presence of spikes during the latter stages of photonic decay (Figure S1C). These observations were consistent with the concomitant detection of large fractions of material which were out of range of the instrumentation (*ca* 36, 51, and 95% respectively).

In order to mitigate the issues experienced when analyzing the native materials, adjuvants were diluted to 1 mg/mL Al using ultrapure water. In all cases, reducing the concentration of aluminum within samples practically abolished non-random sedimentation events (Figure S1D) and increased the amount of particles within range of the instrumentation to >93%. The PDI of Alhydrogel® and Adju-Phos® was also reduced from 1 ± 0 and 0.86 ± 0.07 to 0.20 ± 0.02 and 0.19 ± 0.02 respectively. Dilution prompted a decrease in the median size of all adjuvants studied (1257 ± 54, 2054 ± 68, and 3633 ± 690 nm, *P* = 0.0079, ≤0.0001, and 0.0052 respectively) and this was most evident in the case of Adju-Phos® whose d50 value decreased to reflect that obtained for Alhydrogel® (*P* = 0.10) (Figure 1B and Figure S1B). Imject alum®, however, remained significantly larger than Alhydrogel® under these conditions (*P* = 0.0043) but similar in size to Adju-Phos® (*P* = 0.87).

Alhydrogel® was positively charged in its native form (10.96 ± 0.50 mV) while Adju-Phos® and Imject alum® were both negatively charged, although the latter was closer to neutral (−22.80 ± 3.07 and −0.07 ± 0.38 mV respectively) (Figure 1C). The zeta potential of both Adju-Phos® and Imject alum® decreased upon dilution and the former experienced the greatest shift in magnitude with regards to charge (−38.37 ± 0.52 and −7.47 ± 0.15 mV, *P* = 0.0065 and 0.0079 respectively). By contrast, Alhydrogel® experienced a small but significant increase in zeta potential under these conditions (13.63 ± 0.78 mV, *P* = 0.0079).

### The influence of dilution into saline upon the PSD and zeta potential of aluminum adjuvants

**Figure 2:**
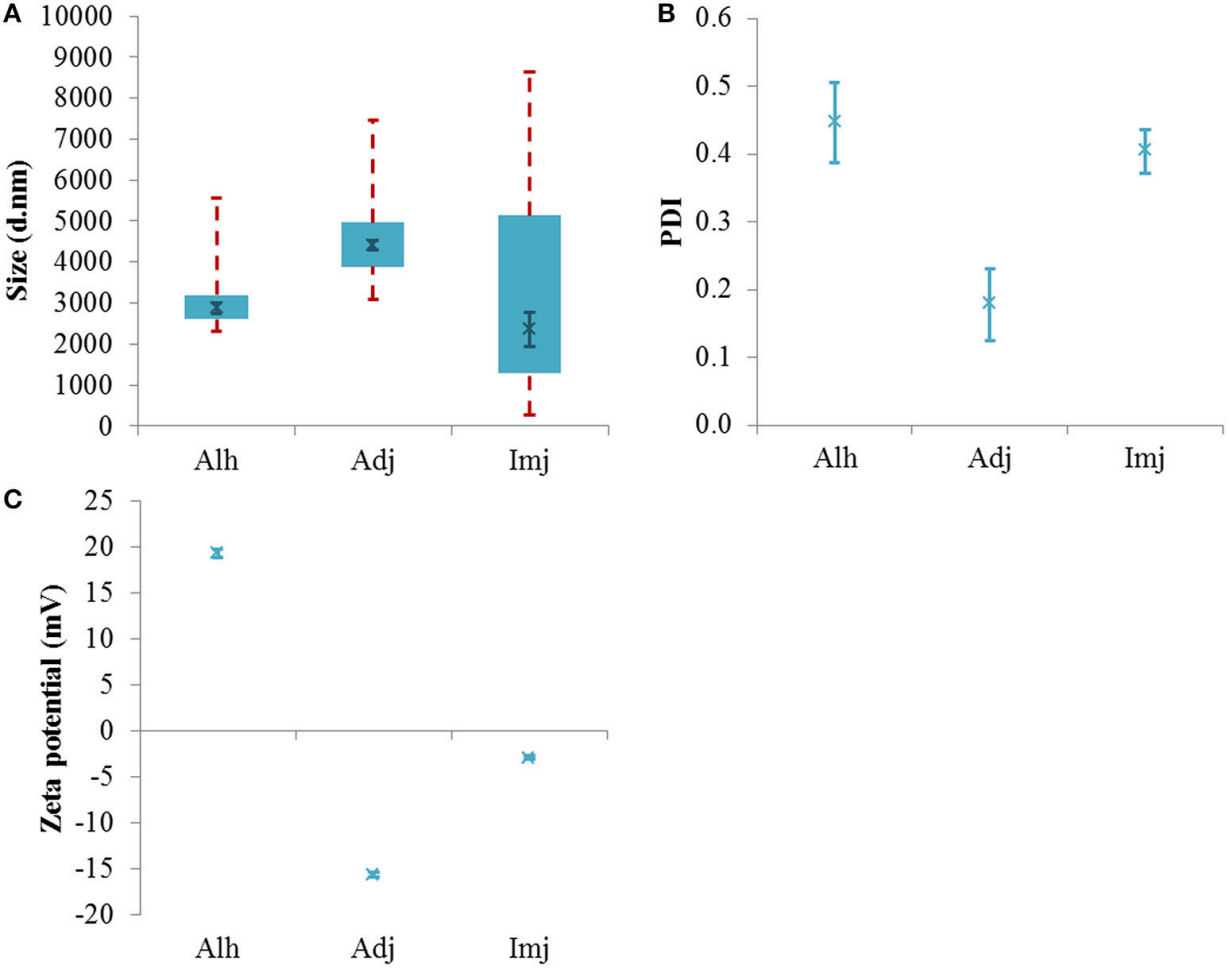
**Particle size distributions (A)**, PDI values **(B)**, and zeta potential **(C)** of aluminum adjuvants prepared in saline to form simulant vaccines containing *ca* 250 μg/mL Al. Box plots are representative of the interquartile range of the data while red dashed lines indicate the maxima and minima. Solid green crosses represent the d50 distribution value (nm). Error bars represent the ±SEM of the measurement where *n* = 5.

The dilution of Alhydrogel® into saline produced a bimodal distribution (Figure S2A) where the majority of scattering was located between 2.6 and 3.2 μm in size (Figure 2A). No difference was observed between the median size of the distribution relative to that observed for the native material (2875 ± 127 nm, *P* = 0.29) despite a noticeable increase in the percentage of detectable particulates (>93%). In comparison to the 1 mg/mL Al solution, however, the d50 value of this vaccine simulant was significantly greater in magnitude (*P* = 0.0079) and an increase in PDI was also noted (0.45 ± 0.059, *P* = 0.012).

The PSD of Adju-Phos® and Imject alum® also exhibited bimodality within this electrolytic environment and the median size of both adjuvants was dramatically reduced when compared to their respective native stocks (4411 ± 128 and 2361 ± 423 nm, *P* = 0.0003 and 0.0001 respectively). The amount of material in range of the sizer also increased to >91 and >93% respectively. The Adju-Phos® vaccine simulant demonstrated a significant increase in median particle size relative to the 1 mg/mL Al stock (*P* ≤ 0.0001); however, the PSD of the Imject alum® simulant remained unaltered (*P* = 0.16). In addition, introduction into saline did not influence the PDI of these distributions when data was compared to that obtained for the respective diluted stocks (0.18 ± 0.053 and 0.40 ± 0.03, *P* = 0.90 and 0.32 respectively) (Figure 2B).

Adjuvant cross-comparisons revealed that particles within the Adju-Phos® simulant were significantly larger than those observed within Alhydrogel® or Imject alum® samples formulated within the same diluent (*P* = 0.0040 and 0.0004 respectively). No difference was observed between the size of the latter materials under these conditions (*P* = 0.39).

All adjuvants maintained the sign of charge exhibited by their respective native stocks when introduced into saline (Figure 2C). While the magnitude of the zeta potential values obtained for both Alhydrogel® and Imject alum® significantly increased and decreased respectively in this diluent (19.33 ± 0.47 and −2.90 ± 0.23 mV, *P* ≤ 0.0001 and 0.008 respectively), no variation in charge was observed when Adju-Phos® was exposed to the same environment (−15.72 ± 0.26 mV, *P* = 0.08). Where comparisons with the 1 mg/mL Al stocks were made, all materials underwent an increase in the magnitude of their zeta potential. (*P* = 0.0079, ≤0.0001, and 0.0079 respectively).

### The influence of protein adsorption upon the PSD and zeta potential of aluminum adjuvants

**Figure 3:**
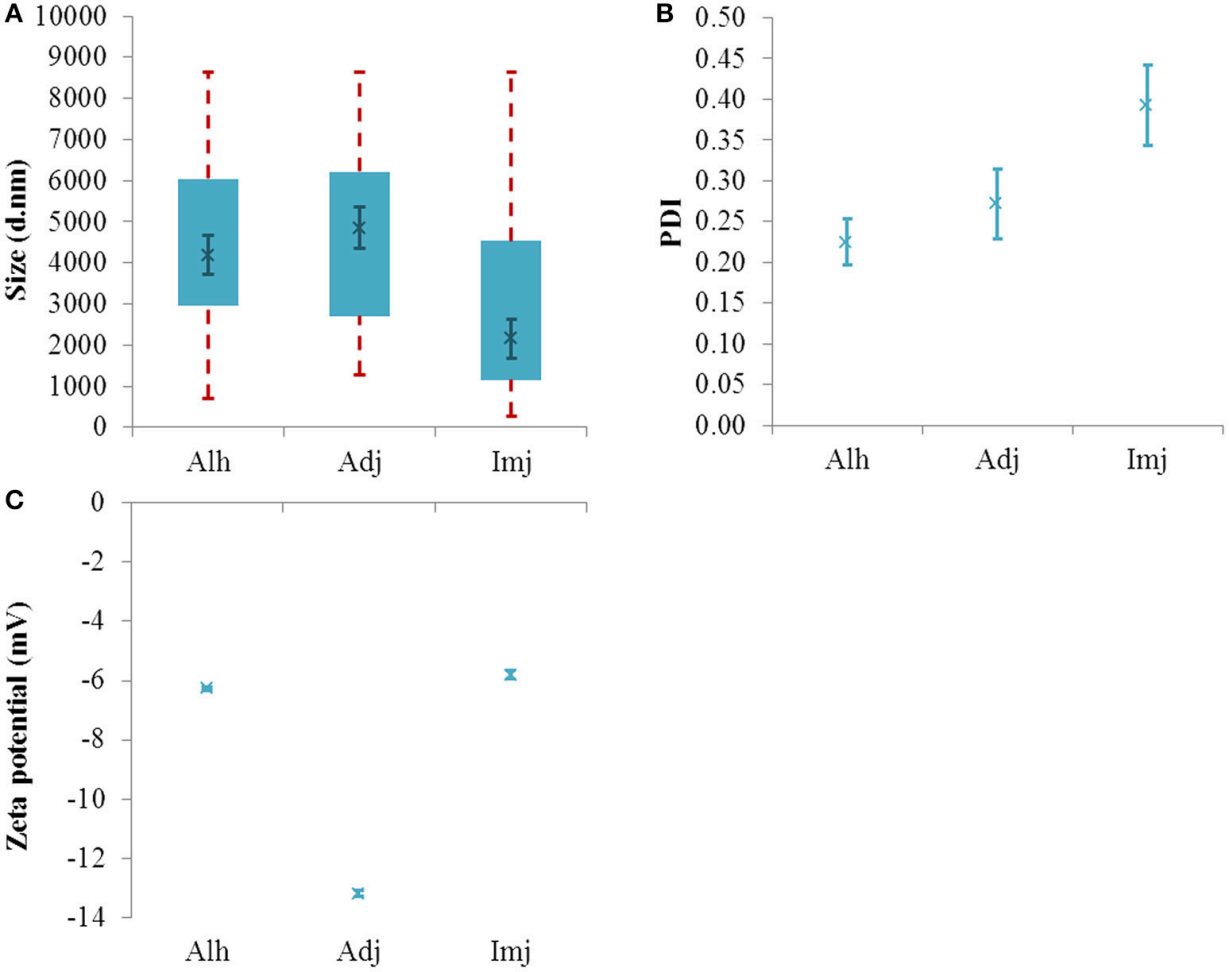
**Particle size distributions (A)**, PDI values **(B)**, and zeta potential **(C)** of aluminum adjuvants formulated in saline + BSA (*ca* 250 μg/mL Al). Box plots are representative of the interquartile range of the data while red dashed lines indicate the maxima and minima. Solid green crosses represent the d50 distribution value (nm). Error bars represent the ±SEM of the measurement where *n* = 5.

The addition of Alhydrogel® to an isotonic system containing BSA not only yielded a substantial increase in median size (4194 ± 466 nm, *P* = 0.045) (Figure 3A) but also a dramatic shift in the value and sign of the adjuvant zeta potential (−6.26 ± 0.06 mV, *p* ≤ 0.0001) (Figure 3C). This was also accompanied by a decrease in PDI (0.22 ± 0.028, *P* = 0.019) (Figure 3B).

Under the same conditions, the PSD of Adju-Phos® remained fairly consistent with the d50 and PDI values showing little deviation from those obtained in saline (4850 ± 501 nm and 0.27 ± 0.04, *P* = 0.44 and 0.21 respectively). In this environment the median size of this adjuvant was similar in magnitude to that obtained for Alhydrogel® (*P* = 0.62). The zeta potential of this material also underwent a small but significant increase relative to the vaccine simulant (−13.02 ± 0.10 mV, *p* = 0.0002).

The PSD of Imject alum® was not influenced by the presence of BSA as demonstrated by the limited variation observed when the median and PDI values were compared against diluent only preparations (2155 ± 485 nm and 0.39 ± 0.05, *P* = 0.76 and 0.85 respectively); however, the zeta potential of the material did decrease in magnitude (−5.81 ± 0.14 mV, *p* ≤ 0.0001).

Where cross comparisons were performed, protein solutions containing Imject alum® generated significantly smaller d50 values than either Alhydrogel® or Adju-Phos® formulations (*P* = 0.029 and 0.0052 respectively).

### The core size of aluminum adjuvants in saline using TEM

**Figure 4:**
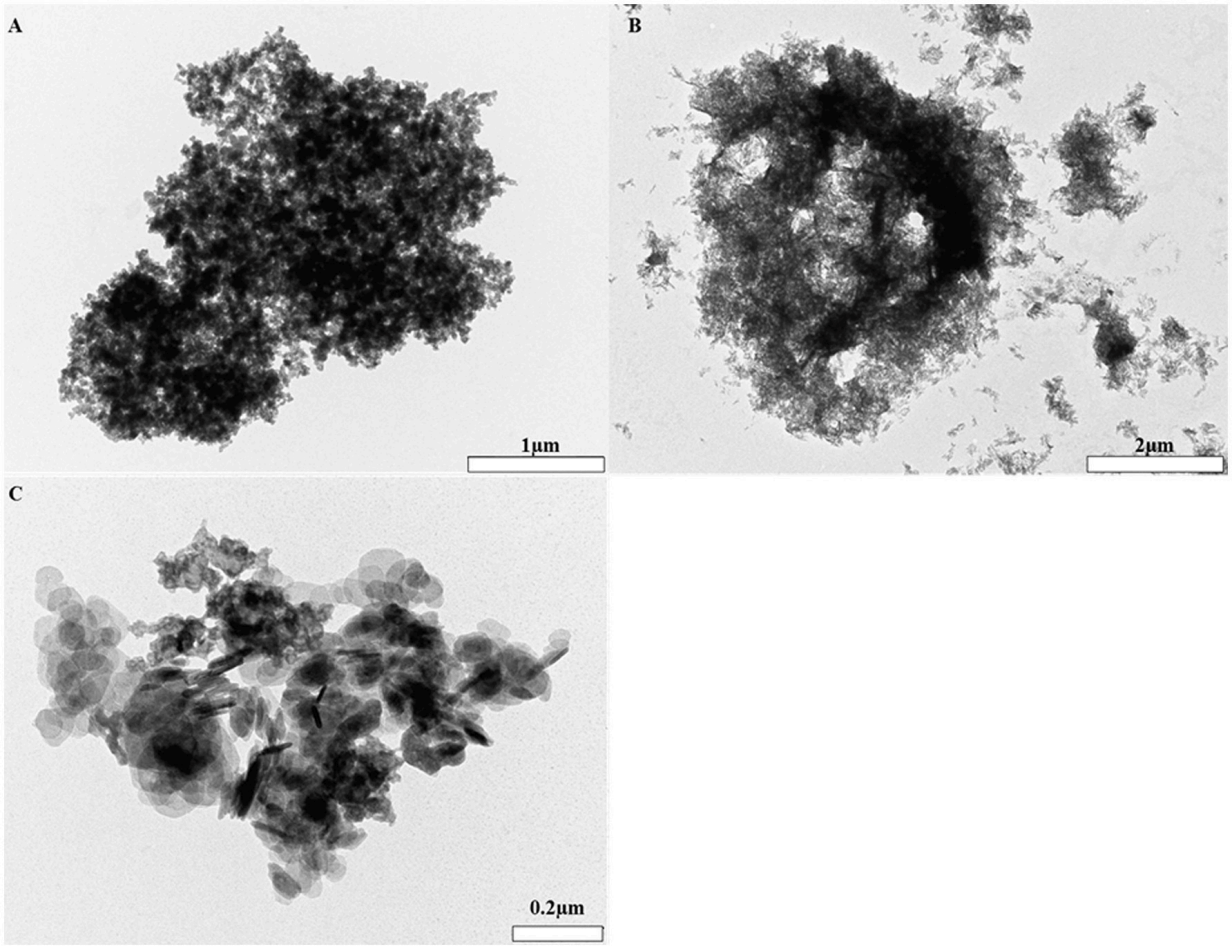
**TEM images of Alhydrogel®** (**B** mag. X30K, scale bar 2 μm), Adju-Phos® (**A** mag. X10K, scale bar 1 μm), and Imject alum® (**C** mag. X100K, scale bar 0.2 μm) formulated in 0.9% NaCl.

Alhydrogel® (Figure 4B) presented as a heterogeneous collection of particulate material where the majority of entities were between *ca* 0.8–2 μm in size. These aggregates were composed of negatively stained primary crystals which were fibrillar in nature. The tendency of such material to self-associate and form large, electron dense agglomerates was also apparent as demonstrated by the presence of a singular structure measuring *ca* 8 μm in size.

Adju-Phos® (Figure 4A) was more homogenous in composition and was predominantly composed of electron dense, porous aggregates whose average sizes equated to *ca* 3 μm. Aggregates were composed of plate-like entities of approximately 50 nm, although this size was subject to variability throughout the structure.

Imject alum® (Figure 4C) was comprised of porous aggregates which were consistently *ca* 1 μm in size and contained two distinct morphologies. The first was composed of several lamellar like crystals and the second reminiscent of the platy morphology observed within Adju-Phos® samples.

## Discussion

Following their industrial fabrication, particle size analysis of adjuvant stocks revealed that a considerable proportion of material within each sample exceeded the maximum limit of detection and was thus unable to be accurately measured using DLS. While this may preclude the derivatization of absolute size values for these materials, application of this methodology provides useful insights into the behavior of these materials in their native environments without the need for further processing or external interference. The propensity of adjuvant particles to self-associate and form agglomerated structures in solution is of particular interest in the study of vaccines and the presence of such entities within stock suspensions has been previously confirmed to some extent using sizing methodologies with a greater upper limit of detection (Burrell et al., 2000b; Lindblad, 2004; Harris et al., 2012). Indeed, the median size obtained herein for Adju-Phos® (7152 ± 308 nm) is in good agreement with that obtained using low angle laser light scattering if typical batch variation is also considered (6.2 μm—Kolade et al., 2015), which adds some legitimacy to both our native stock adjuvant data and approach to sizing despite its noted limitations.

Relative cross-comparisons between adjuvants revealed marked differences between the median size of native materials and their potential to agglomerate. This tendency toward agglomeration appeared to be governed by the interaction of these particles with the surrounding aqueous environment. Adjuvants whose structures were extensively hydrated i.e., Adju-Phos® and Imject alum® (Shirodkar et al., 1990; Hem et al., 2007) yielded the largest median particle size and contained the highest proportion of entities exceeding *ca* 9 μm, which infers that a large amount of particle-solvent interactions may encourage self-association. The degree of colloidal instability experienced within these systems was also consistent with their respective zeta potential readings with the latter being close to neutrality and as a consequence exhibiting the greatest amount of sample agglomeration.

Quite conversely, however, the zeta potential of Alhydrogel® inferred that the agglomeration potential of this adjuvant was greater than that of Adju-Phos®, which was simply not the case. In fact, this material expressed both the lowest abundance of agglomerates and the smallest median particle size, which plausibly may be attributed to its crystalline and less-hydrated structure (Shirodkar et al., 1990). Furthermore, the high polydispersity of this sample in particular calls into question the monomodal nature of its distribution and perhaps gives a preliminary indication of the existence and contribution of particulates well in excess of the *ca* 9 μm maxima. Indeed, other studies have identified several particulate populations within colloidal aluminum oxyhydroxide solutions which exceed this limit including one at 22 μm and another at 44 μm (Wolff et al., 2008). It follows then that despite its lower d50 and out of range values, Alhydrogel® may contain some of the largest singular agglomerates. There is some evidence to suggest that the presence of the latter may be related to a lack of superficial hydrogen bonding within suspension, which has also been shown to facilitate the nucleation and growth of colloids (Banfield et al., 2000). When the observations for both the amorphous and crystalline native adjuvants are considered, such information implies that an optimal amount of adjuvant-diluent interactions are required to minimize adjuvant agglomeration, a characteristic which can negatively impact the efficacy of vaccines (Maa et al., 2003; Clausi et al., 2008; Braun et al., 2009; Chen et al., 2009).

The PSD of aluminum adjuvants is also heavily influenced by dilution, which acts to increase the separation of particles in solution and thus reduce the likelihood of aggregate formation. Significant disaggregation was observed within all the colloidal suspensions studied when the concentration of aluminum was reduced with the PSD of Adju-Phos® being the most susceptible to these events. This correlated with the concomitant shifts in adjuvant surface charge toward more negative or positive values of zeta potential, insinuating that contributions from repulsive forces were increasing and thus acting to stabilize the size of particulates. Unlike the mechanical separation induced by interfacial shear, it remains unclear whether these repulsive forces are strong enough to maintain stability in the long-term, especially in the case of Alhydrogel® and Imject alum® whose zeta potential readings highlighted that some degree of systemic instability was still present within these solutions. From a biological perspective, reducing the propensity of self-association through modification of adjuvant dosing may be beneficial with regards to maximizing particle phagocytosis and systemic translocation by immune cells. In line with the results of this study, vaccine simulants prepared using lower doses of aluminum were shown to contain smaller aggregates than their more concentrated counterparts and as a consequence demonstrated an elevated cerebral presence following injection (Crépeaux et al., 2016).

The vast majority of vaccines are formulated in physiological saline and contain between 125 and 800 μg/mL Al (Baylor et al., 2002). In this isotonic environment, whose matrix is teaming with monovalent electrolytes, charge screening allows interactive van der Waal forces between adjuvant particles to predominate, which in theory should facilitate both aggregation and agglomeration (Derjaguin and Landau, 1941; Verwey and Overbeek, 1948). Indeed, commercial preparations experienced varying degrees of aggregation when introduced into this environment when size comparisons were made with their respective diluted adjuvant stocks. Surprisingly, these events did not necessarily correlate with the expected shift in zeta potential and quite conversely the surface charge of Alhydrogel® actively increased in this medium, a trend associated with heightened contributions from electrostatic repulsion and thus enhanced colloidal stability.

During the formulation of vaccines, however, it is the native concentrated stock which is directly mixed with the diluent and discussing the impact of these events upon the PSD of aluminum adjuvants is perhaps more relevant in this context. When mimicked experimentally, the median size of all materials, exclusive of Alhydrogel®, underwent a significant reduction. A dilution of this magnitude did, however, result in the abolition of the large agglomerates observed within the native Alhydrogel® stock. It can therefore be argued that the removal/disaggregation of these particles also constitutes a large reduction in particle size and results in an increase in systemic stability, which is consistent with the increase in zeta potential observed. Furthermore, it is clear that when both dilution and ionic strength are considered within the same system, it is particulate disaggregation that is favored during the initial stages of addition and the influence of dilution is dominates.

The degree to which proteins are adsorbed by aluminum salts is another factor that governs the size of the particles found within vaccine preparations. Several studies have reported substantial adsorption capacities and coefficients for the Alhydrogel®/BSA complex while at the same time observing virtually no adsorption of this protein by Adju-Phos® (Seeber et al., 1991; Al-Shakhshir et al., 1994; Jones et al., 2005). Others have also commented upon the poor antigen adsorption capacity of Imject alum® and proposed this as reasoning for the limited immunological efficacy of this adjuvant *in vivo* (Cain et al., 2013). In our study, the masking of native particle charge observed within Alhydrogel® model vaccines inferred that this adjuvant exhibited a high degree of BSA surface coverage, which can be attributed to the favorable electrostatic interactions between the protein and adjuvant (Al-Shakhshir et al., 1994). These results suggest that it was this degree of protein adsorption that was directly associated with the significant elevation in particle size observed relative to the simulant only preparations. In the case of Adju-Phos® and Imject alum®, zeta potential results also suggested that some adsorption had occurred at the colloidal interface of these materials. However, due to the small magnitude of these variations, it is likely that electrostatic repulsion still remained dominant within these systems, which in all probability severely restricted protein adsorption. This is consistent with the null significance observed between the size of these adjuvants in saline vs. that of the antigen-adjuvant complexes formed in model vaccines.

The size and consequent recognition of the adjuvant-antigen complex is thus likely to be dependent on: (i) The species of protein used as an antigen and (ii) The magnitude of the association constant between adjuvant and antigen. High affinity combinations are likely to experience less antigenic elution when exposed to the physiological milieu at the injection site (Noe et al., 2010) and thus retain their enhanced size. The latter may serve to optimize the recognition and uptake of these complexes as well as delivering a greater amount of antigen for cellular processing, which in turn improves the kinetics and efficacy of the immunological response (Mannhalter et al., 1985). In our recent communication, we demonstrated high levels of adjuvant loading within the cytoplasm of THP-1 cells exposed to the Alhydrogel®-BSA complexes formed through the exposure of the adjuvant to nutrient rich culture medium (Mold et al., 2016). This observation was attributed to the large number of particles falling within the optimal size range for macrophagic recognition, which is reportedly between 2 and 3 μm (Champion et al., 2008). However, strong associative forces between adjuvant and antigen can also induce the formation of agglomerates exceeding 10 μm (Morefield et al., 2005) and weak adsorption coefficients have been associated with the generation of higher relative antibody titres (Iyer et al., 2003; Hansen et al., 2007, 2009; Noe et al., 2010). Such agglomerates prove challenging to engulf (Morefield et al., 2005; Mold et al., 2016) and their subsequent cellular translocation to the lymph nodes may be significantly impeded as a result. In addition, conformational changes induced upon the adsorption of protein are more frequently encountered at the low concentrations of antigen used herein and in clinical vaccines (Lassen and Malmsten, 1996; Rabe et al., 2011). These alterations in protein structure have been linked to a decrease in particle-cellular adhesion and a subsequent reduction in their internalization by phagocytes (Yan et al., 2013); however, similar patterns of behavior have not yet been observed in the case of aluminum salts.

As alluded to at the beginning of this discussion, it is acknowledged that the limitations of DLS make the accurate characterization of polydisperse and agglomerated solutions challenging. The random movement of particles within highly concentrated solutions can be severely restricted and the intensity based nature of the measurements precludes the objective analysis of sample diversity. The latter was observed through the complementary use of microscopy but this technique requires conditions which are non-hydrodynamic and as a consequence particulate dimensions are comparatively smaller. It is thus not unreasonable to suggest that the biological reactivity of these materials will be more dependent on the abundance of these discrete populations, which may also include the presence of metal ions. Future studies will therefore focus upon the characterization and quantification of these individual populations in order to establish their relative physiological impact.

## Conclusion

While interest in the physicochemical characterization of aluminum salts has waned over recent years, such studies are crucial with regards to the development of safer and more effective vaccines. The link between particle size and immunopotentiation, although conflicting, has been widely established (Xiang et al., 2006; Oyewumi et al., 2010) but still there remains a limited body of work in this area specifically relating to aluminum adjuvants. To this effect, this is the first study to directly monitor the hydrodynamic PSD of a variety of aluminum-based adjuvants over the systematic stages of vaccine preparation. We have shown herein that the particle size of aluminum salts is heavily influenced by the concentration of aluminum and degree of protein adsorption experienced within the vaccine preparation itself during the formulation process. More importantly, it is likely that the resultant size of these antigen-adjuvant complexes will in some way dictate their subsequent recognition and translocation by components of the immune system.

## Author contributions

ES, CE, and MM contributed toward the study design. ES performed all particle size and zeta potential measurements of aluminum adjuvant preparations, prepared all figures, and wrote the manuscript. MM performed the TEM imaging. All authors read and commented upon the manuscript before submission.

## Funding

This study was supported by funding from the CMSRI (Children's Medical Safety Research Institute).

### Conflict of interest statement

The authors declare that the research was conducted in the absence of any commercial or financial relationships that could be construed as a potential conflict of interest.

## References

[B1] Al-Shakhshir R. Regnier F. White J. L. Hem S. L. (1994). Effect of protein adsorption on the surface charge characteristics of aluminium-containing adjuvants. Vaccine 12, 472–474. 10.1016/0264-410X(94)90127-9 8023556

[B2] Banfield J. F. Welch S. A. Zhang H. Ebert T. T. Penn R. L. (2000). Aggregation-based crystal growth and microstructure development in natural iron oxyhydroxide biomineralization products. Science 289, 751–754. 10.1126/science.289.5480.751 10926531

[B3] Baylor N. W. Egan W. Richman P. (2002). Aluminium salts in vaccines – US perspective. Vaccine 29, S18–S23. 10.1016/S0264-410X(02)00166-4 12184360

[B4] Braun L. J. Tyagi A. Perkins S. Carpenter J. Sylvester D. Guy M.. (2009). Development of a freeze-stable formulation for vaccines containing aluminum salt adjuvants. Vaccine 27, 72–79. 10.1016/j.vaccine.2008.10.027 18973782

[B5] Brewer J. M. Conacher M. Satoskar A. Bluethmann H. Alexander J. (1996). In interleukin-4-deficient mice, alum not only generates T helper 1 responses equivalent to freund's complete adjuvant, but continues to induce T helper 2 cytokine production. Eur. J. Immunol. 26, 2062–2066. 10.1002/eji.1830260915 8814247

[B6] Burrell L. S. Johnston C. T. Schulze D. Klein J. White J. L. Hem S. L. (2000a). Aluminium phosphate adjuvants prepared by precipitation at constant pH. Part II: physicochemical properties. Vaccine 19, 282–287. 10.1016/S0264-410X(00)00162-6 10930683

[B7] Burrell L. S. White J. L. Hem S. L. (2000b). Stability of aluminium-containing adjuvants during aging at room temperature. Vaccine 18, 2188–2192. 10.1016/S0264-410X(00)00031-1 10717337

[B8] Cain D. W. Sanders S. E. Cunningham M. M. Kelsoe G. (2013). Disparate adjuvant properties among three formulations of “alum.” Vaccine 31, 653–660. 10.1016/j.vaccine.2012.11.044 23200935 PMC3541451

[B9] Champion J. A. Walker A. Mitragotri S. (2008). Role of particle size in phagocytosis of polymeric microspheres. Pharm. Res. 25, 1815–1821. 10.1007/s11095-008-9562-y 18373181 PMC2793372

[B10] Chen D. Tyagi A. Carpenter J. Perkins S. Sylvester D. Guy M.. (2009). Characterization of the freeze sensitivity of a hepatitis B vaccine. Hum. Vaccin. 5, 26–32. 10.4161/hv.5.1.6494 18971625

[B11] Clausi A. Cummiskey J. Merkley S. Carpenter J. F. Braun L. J. Randolph T. W. (2008). Influence of particle size and antigen binding on effectiveness of aluminum salt adjuvants in a model lysozyme vaccine. J. Pharm. Sci. 97, 5252–5262. 10.1002/jps.21390 18398901

[B12] Cox J. Coulter A. (1997). Adjuvants - a classification and review of their modes of action. Vaccine 15, 248–256. 10.1016/S0264-410X(96)00183-1 9139482

[B13] Crépeaux G. Eidi H. David M. O. Giros B. Authier F. J. Exley C. (2016). Low concentrations of aluminum hydroxide adjuvant, forming limited size aggregates, selectively induce cerebral aluminum increase and long-term neurotoxicity in mouse. Morphologie 100, 160–161. 10.1016/j.morpho.2016.07.003

[B14] Derjaguin B. Landau L. (1941). Theory of the stability of strongly charged lyophobic sols and of the adhesion of strongly charged particles in solution of electrolytes. *Acta. Physicochim*. URSS. 14, 633–662.

[B15] Eisenbarth S. C. Colegio O. R. O'Connor W. Sutterwala F. S. Flavell R. A. (2008). Crucial role for the Nalp3 inflammasome in the immunostimulatory properties of aluminium adjuvants. Nature 453, 1122–1126. 10.1038/nature06939 18496530 PMC4804622

[B16] Exley C. Siesjö P. Eriksson H. (2010). The immunobiology of aluminium adjuvants: how do they really work? Trends Immunol. 31, 103–109. 10.1016/j.it.2009.12.009 20153253

[B17] Gherardi R. K. Coquet M. Cherin P. Belec L. Moretto P. Dreyfus P. A.. (2001). Macrophagic myofasciitis lesions assess long-term persistence of vaccine-derived aluminium hydroxide in muscle. Brain 124, 1821–1831. 10.1093/brain/124.9.1821 11522584

[B18] Goldenthal K. L. Cavagnaro J. A. Alving C. Vogel F. R. (1993). Safety evaluation of vaccine adjuvants. NCVDG working groups. *AIDS Res*. Hum. Retroviruses 9, s47–s51.

[B19] Grun J. L. Maurer P. H. (1989). Different T helper cell subsets elicited in mice utilizing two different adjuvant vehicles: the role of endogenous interlukin-1 in proliferative responses. Cell Immunol. 121, 134–145. 10.1016/0008-8749(89)90011-7 2524278

[B20] Gupta R. K. (1998). Aluminum compounds as vaccine adjuvants. Adv. Drug. Deliv. Rev. 32, 155–172. 10.1016/S0169-409X(98)00008-8 10837642

[B21] Hansen B. Belfast M. Soung G. Song L. Egan P. M. Capen R.. (2009). Effect of the strength of adsorption of hepatitis B surface antigen to aluminum hydroxide adjuvant on the immune response. Vaccine 27, 888–892. 10.1016/j.vaccine.2008.11.078 19071182

[B22] Hansen B. Sokolovska A. HogenEsch H. Hem S. L. (2007). Relationship between the strength of antigen adsorption to an aluminum-containing adjuvant and the immune response. Vaccine 25, 6618–6624. 10.1016/j.vaccine.2007.06.049 17681647

[B23] Harris J. R. Soliakov A. Lewis R. J. Depoix F. Watkinson A. Lakey J. H. (2012). Alhydrogel® adjuvant, ultrasonic dispersion and protein binding: a TEM and analytical study. Micron 43, 192–200. 10.1016/j.micron.2011.07.012 21831642

[B24] Hem S. L. Johnston C. T. HogenEsch H. (2007). Imject Alum is not aluminum hydroxide adjuvant or aluminum phosphate adjuvant. Vaccine 25, 4985–4986. 10.1016/j.vaccine.2007.04.078 17543429

[B25] Huang M. Wang W. (2014). Factors affecting alum-protein interactions. Int. J. Pharm. 466, 139–146. 10.1016/j.ijpharm.2014.03.015 24607202

[B26] Iyer S. HogenEsch H. Hem S. L. (2003). Relationship between the degree of antigen adsorption to aluminum hydroxide adjuvant in interstitial fluid and antibody production. Vaccine 21, 1219–1223. 10.1016/S0264-410X(02)00556-X 12559801

[B27] Iyer S. Robinett R. S. HogenEsch H. Hem S. L. (2004). Mechanism of adsorption of hepatitis B surface antigen by aluminum hydroxide adjuvant. Vaccine 22, 1475–1479. 10.1016/j.vaccine.2003.10.023 15063571

[B28] Jones L. S. Peek L. J. Power J. Markham A. Yazzie B. Middaugh C. R. (2005). Effects of adsorption to aluminum salt adjuvants on the structure and stability of model protein antigens. J. Biol. Chem. 280, 13406–13414. 10.1074/jbc.M500687200 15684430

[B29] Jordan M. B. Mills D. M. Kappler J. Marrack P. Cambier J. C. (2004). Promotion of B cell immune responses via an alum-induced myeloid cell population. Science 304, 1808–1810. 10.1126/science.1089926 15205534

[B30] Kolade O. O. Jin W. Tengroth C. Green K. D. Bracewell D. G. (2015). Shear effects on aluminum phosphate adjuvant particle properties in vaccine drug products. J. Pharm. Sci. 104, 378–387. 10.1002/jps.24127 25175154

[B31] Lassen B. Malmsten M. (1996). Structure of protein layers during competitive adsorption. J. Colloid. Interface Sci. 180, 339–349. 10.1006/jcis.1996.0312

[B32] Lindblad E. B. (2004). Aluminium compounds for use in vaccines. Immunol. Cell Biol. 82, 497–505. 10.1111/j.0818-9641.2004.01286.x 15479435

[B33] Maa Y. F. Zhao L. Payne L. G. Chen D. (2003). Stabilization of alum-adjuvanted vaccine dry powder formulations: mechanism and application. J. Pharm. Sci. 92, 319–332. 10.1002/jps.10294 12532382

[B34] Mannhalter J. W. Neychev H. O. Zlabinger G. J. Ahmad R. Eibl M. M. (1985). Modulation of the human immune response by the non-toxic and non-pyrogenic adjuvant aluminium hydroxide: effect on antigen uptake and antigen presentation. Clin. Exp. Immunol. 61, 143–151. 3876178 PMC1577243

[B35] Mold M. Ouro-Gnao L. Wieckowski B. Exley C. (2013). Copper prevents amyloid-β1–42 from forming amyloid fibrils under near-physiological conditions *in vitro*. Sci. Rep. 3, 1256–1262. 10.1038/srep01256 23409247 PMC3570782

[B36] Mold M. Shardlow E. Exley C. (2016). Insight into the cellular fate and toxicity of aluminium adjuvants used in clinically approved human vaccinations. Sci. Rep. 6:31578. 10.1038/srep31578 27515230 PMC4981857

[B37] Morefield G. L. Sokolovska A. Jiang D. HogenEsch H. Robinson J. P. Hem S. L. (2005). Role of aluminum-containing adjuvants in antigen internalization by dendritic cells *in vitro*. Vaccine 23, 1588–1595. 10.1016/j.vaccine.2004.07.050 15694511

[B38] Noe S. M. Green M. A. HogenEsch H. Hem S. L. (2010). Mechanism of immunopotentiation by aluminum-containing adjuvants elucidated by the relationship between antigen retention at the inoculation site and the immune response. Vaccine 28, 3588–3594. 10.1016/j.vaccine.2010.02.085 20211692

[B39] Oyewumi M. O. Kumar A. Cui Z. (2010). Nano-microparticles as immune adjuvants: correlating particle sizes and the resultant immune responses. Expert Rev. Vaccines 9, 1095–1107. 10.1586/erv.10.89 20822351 PMC2963573

[B40] Rabe M. Verdes D. Seeger S. (2011). Understanding protein adsorption phenomena at solid surfaces. Adv. Colloid Interface Sci. 162, 87–106. 10.1016/j.cis.2010.12.007 21295764

[B41] Reed S. G. Orr M. T. Fox C. B. (2013). Key roles of adjuvants in modern vaccines. Nat. Med. 19, 1597–1608. 10.1038/nm.3409 24309663

[B42] Seeber S. J. White J. L. Hem S. L. (1991). Predicting the adsorption of proteins by aluminium-containing adjuvants. Vaccine 9, 201–203. 10.1016/0264-410X(91)90154-X 2042392

[B43] Shah R. R. O'Hagan D. T. Amiji M. M. Brito L. A. (2014). The impact of size on particulate vaccine adjuvants. Nanomedicine 9, 2671–2681. 10.2217/nnm.14.193 25529570

[B44] Shaw C. A. Petrick M. S. (2009). Aluminum hydroxide injections lead to motor deficits and motor neuron degeneration. J. Inorg. Biochem. 103, 1555–1562. 10.1016/j.jinorgbio.2009.05.019 19740540 PMC2819810

[B45] Shirodkar S. Hutchinson R. L. Perry D. L. White J. L. Hem S. L. (1990). Aluminium compounds used as adjuvants in vaccines. Pharm. Res. 7, 1282–1288. 10.1023/A:1015994006859 2095567

[B46] Shoenfeld Y. Agmon-Levin N. (2011). Autoimmune/inflammatory syndrome induced by adjuvants. J. Autoimmun. 36, 4–8. 10.1016/j.jaut.2010.07.003 20708902

[B47] Verwey E. J. W. Overbeek J. Th. G. (1948). Theory of the Stability of Lyophobic Colloids: The Interaction of Sols Particles Having an Electric Double Layer. Amersterdam: Elsevier Inc.

[B48] Wolff L. Flemming J. Schmitz R. Gröger K. Müller-Goymann C. (2008). Protection of aluminum hydroxide during lyophilisation as an adjuvant for freeze-dried vaccines. Colloids Surfaces A Physicochem. Eng. Aspects 330, 116–126. 10.1016/j.colsurfa.2008.07.031

[B49] Xiang S. D. Scholzen A. Minigo G. David C. Apostolopoulos V. Mottram P. L.. (2006). Pathogen recognition and development of particulate vaccines: does size matter? Methods 40, 1–9. 10.1016/j.ymeth.2006.05.016 16997708

[B50] Yan Y. Gause K. T. Kamphuis M. M. Ang C. S. O'Brien-Simpson N. M. Lenzo J. C.. (2013). Differential roles of the protein corona in the cellular uptake of nanoporous polymer particles by monocyte and macrophage cell lines. ACS Nano 7, 10960–10970. 10.1021/nn404481f 24256422

