# Supplementary material for: From Stock Bottle to Vaccine: Elucidating the Particle Size Distributions of Aluminum Adjuvants Using Dynamic Light Scattering
Source: Front Chem. 2017 Jan 9;4:48. doi: 10.3389/fchem.2016.00048 (PMC5220009; doi:10.3389/fchem.2016.00048)
Supplement: Supplementary file 1 [file DataSheet1.DOCX]

Supplementary Material

**From stock bottle to vaccine: Elucidating the particle size distributions of aluminum adjuvants using dynamic light scattering.**

Emma Shardlow^1^, Matthew Mold^1^, Christopher Exley^1*^

^1^The Birchall Centre, Lennard-Jones Laboratories, Keele University, Keele, Staffordshire, ST5 5BG, UK.

*** Correspondence:**Corresponding Author
c.exley@keele.ac.uk

# Supplementary Data


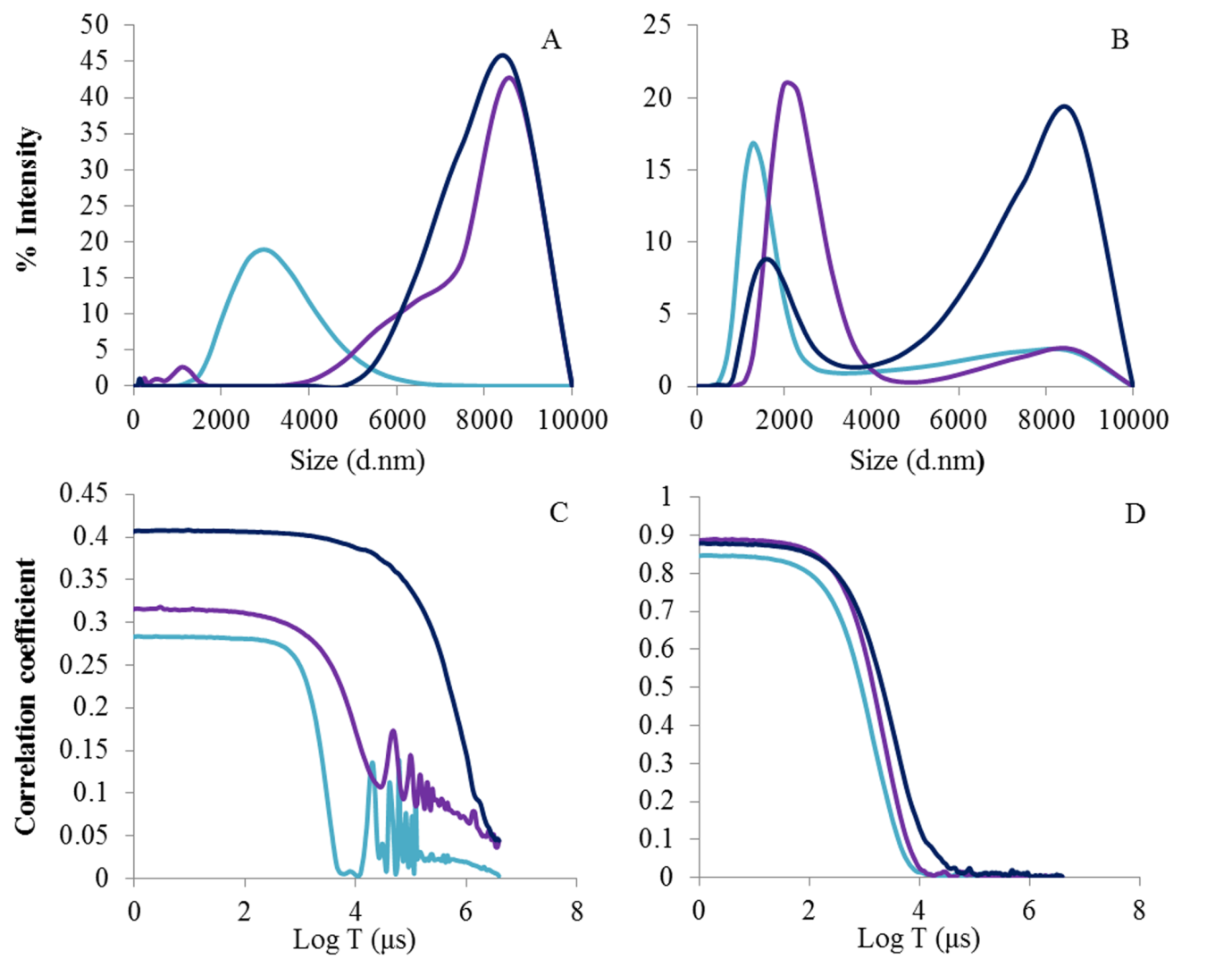


Fig. S1: Raw intensity distributions generated for native aluminum adjuvants (a) and those diluted to 1mg/mL Al in UPW (b). Green, purple and blue lines indicate results obtained for Alhydrogel^®^, Adju-Phos^®^ and Imject alum^®^ respectively. Their respective correlation coefficients are presented in figures c & d.


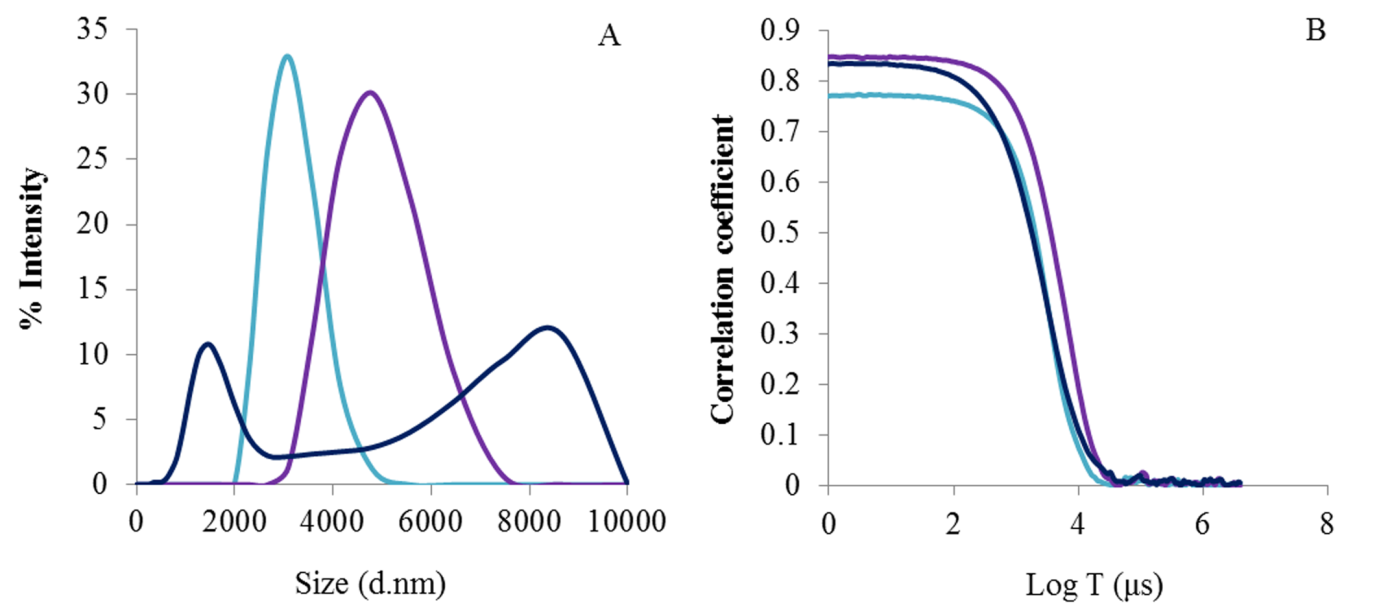


Fig. S2: Raw intensity distributions generated for aluminum adjuvants formulated in 250μg/mL NaCl (a) and their respective correlation coefficients (b). Green, purple and blue lines indicate results obtained for Alhydrogel^®^, Adju-Phos^®^ and Imject alum^®^ respectively.


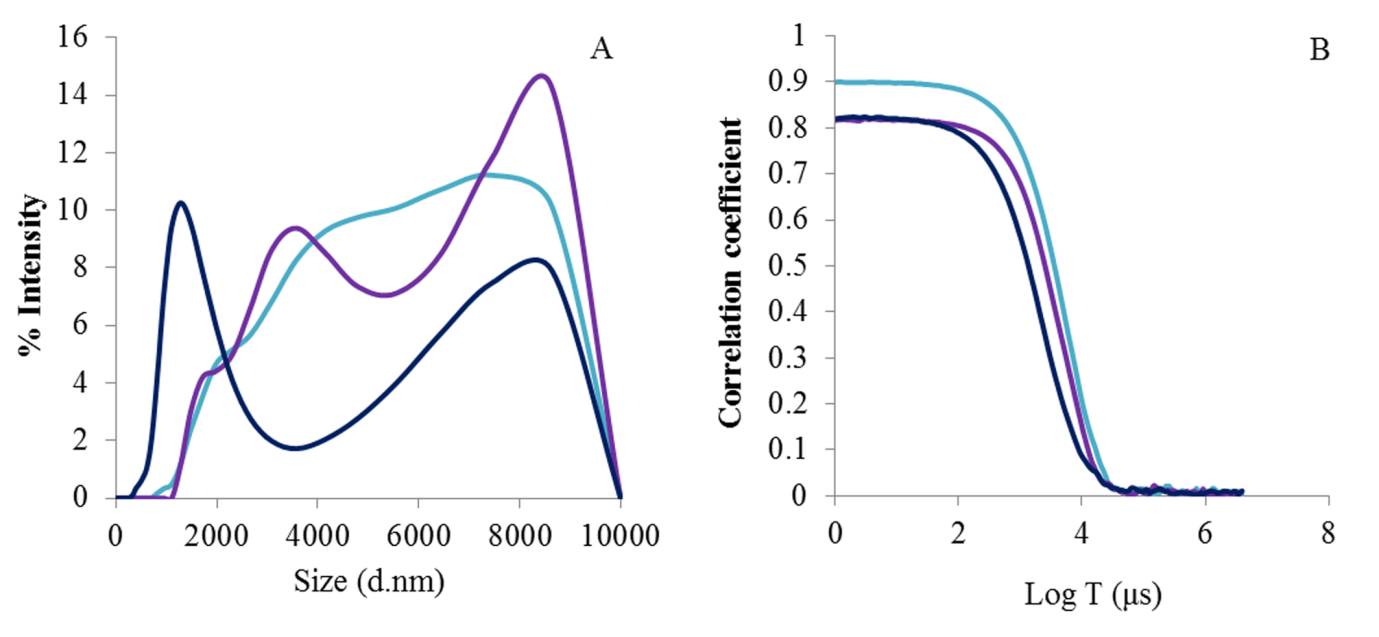


Fig. S3: Raw intensity distributions generated for aluminum adjuvants formulated in NaCl + BSA (a) and their respective correlation coefficients (b). Green, purple and blue lines indicate results obtained for Alhydrogel^®^, Adju-Phos^®^ and Imject alum^®^ respectively.
